# Design of Sb_2_Te_3_ nanoblades serialized by Te nanowires for a low-temperature near-infrared photodetector

**DOI:** 10.3389/fchem.2022.1060523

**Published:** 2022-11-18

**Authors:** Hong Yin, Huaiyu Li, Xiang-xiang Yu, Minglei Cao

**Affiliations:** ^1^ School of Chemistry and Chemical Engineering, Hunan Institute of Science and Technology, Yueyang, China; ^2^ International Iberian Nanotechnology Laboratory (INL), Braga, Portugal; ^3^ School of Physics and Optoelectronic Engineering, Yangtze University, Jingzhou, China; ^4^ School of Mathematics, Physics and Optoelectronic Engineering, Hubei University of Automotive Technology, Shiyan, China

**Keywords:** photodetector, responsivity and detectivity, low-temperature, heterojunction, epitaxial growth

## Abstract

The dangling bond on the surface of bulk materials makes it difficult for a physically contacted heterojunction to form an ideal contact. Thus, periodic epitaxial junctions based on Sb_2_Te_3_ nanoblades serialized by Te nanowires (Sb_2_Te_3_/Te) were fabricated using a one-step hydrothermal epitaxial growth method. X-ray diffraction and electron microscopy reveal that the as-prepared product has a good crystal shape and heterojunction construction, which are beneficial for a fast photoresponse due to the efficient separation of photogenerated carriers. When the Sb_2_Te_3_/Te composite is denoted as a photodetector, it shows superior light response performance. Electrical analysis showed that the photocurrent of the as-fabricated device declined with temperatures rising from 10K to 300K at 980 nm. The responsivity and detectivity were 9.5 × 10^11^ μA W^−1^ and 1.22 × 10^11^ Jones at 50 K, respectively, which shows better detection performance than those of other Te-based photodetector devices. Results suggest that the as-constructed near-infrared photodetector may exhibit prospective application in low-temperature photodetector devices.

## Introduction

Topological insulators have been experiencing new grading of quantum matter consisting of a bulk gap and Dirac-like surface states (Fu et al., 2007; Xia et al., 2009). These materials were considered using a robust spin-orbit interaction that leads to surface states bridging the bulk band gap. More importantly, the carriers on the surface states of topological insulators have low energy dissipation because of the time-reversal symmetry and spin-orbit coupling (Yu et al., 2018). Moreover, angle-resolved photoemission spectroscopy (ARPES) analysis indicates that the surface states consist of an odd number of helical spin-momentum textured Dirac cones (Pradhan et al., 2017; Sun et al., 2017). Therefore, methods have been developed to synthesize various topological insulator materials, for example, metal-organic chemical vapor deposition (MOCVD), pulsed-laser deposition (PLD), and physical vapor deposition (PVD) (Jiang et al., 2005; Jin et al., 2005; Matsunaga et al., 2006; Ikeda et al., 2007). Due to unique physical properties and potential applications in more and more fields, such as quantum computing, photodetection, and superconductors, topological insulator materials have been the focus of tremendous recent attention (Duan et al., 2015; Yu et al., 2017). In these devices, photodetectors have gained special attention because of their widespread applications in many areas, such as industrial automatic control, infrared remote sensing, image sensors, and target detection (Yang et al., 2000; Matsunaga et al., 2004; Zhong et al., 2017; Wang et al., 2022). For example, a photoconductor based on topological insulator (Sb_2_Te_3_) film has been prepared, and the device has the ability to detect the 980 nm near-infrared light (Zheng et al., 2015). Zhang et al. reported a polycrystalline Bi_2_Te_3_ film topological insulator for a near-infrared (NIR) photodetector and revealed that the as-prepared device is sensitive to visible and NIR light and the responsivity and gain are 3.3 × 10^−5^ A W^−1^ and 3.85 × 10^−5^, respectively (Wesolowski et al., 2014). In addition, a series of photovoltaic detectors based on topological insulators, such as SnTe/Si and Sb_2_Te_3_/STO, were prepared and exhibited excellent performance (Tominaga et al., 2014; Sun et al., 2017). However, these devices exhibit a large dark current and the very low carrier lifetime of the photoconductor based on one component leads to a slower response speed and little photocurrent.

Sb_2_Te_3_, as a narrow bandgap semiconductor (∼0.23 eV), is considered a rhombohedral crystal showing a bulky periodicity along its *c*-axis (*a*_Sb2Te3_ = 4.26 Å, *c*_Sb2Te3_ = 30.46 Å). Additionally, Tellurium is a key semiconductor and its bandgap is approximately 0.35 eV (Cheng et al., 2013; Li et al., 2015). Therefore, Sb_2_Te_3_ and Te nanostructures can be epitaxially grown together to form heterojunctions due to the similarity of lattice spacings. Factually, the enhanced concentration of interfaces can strongly boost the formation of the built-in field. The effect is beneficial for separating photon-induced carriers (Lee et al., 2008; Tominaga et al., 2008; Sosso et al., 2009; Dong et al., 2010). Consequently, photodetection performances would be enhanced due to the quantum size effects by forming heterojunctions (Chen et al., 2003; Cozzoli et al., 2006).

Although the synthesis of various Sb_2_Te_3_-Te heterojunctions has been executed and performance has been estimated, the complex and energy-intensive fabrication process of the heterojunction, such as CVD and MBE, seriously restricts their large-scale application. Herein, we settled on a facile one-step hydrothermal method to prepare T-shaped epitaxial Sb_2_Te_3_/Te heterojunctions with feature sizes of hundreds of nanometers. The morphologies and structures of the product were carefully characterized by an electron microscope and X-ray diffraction, respectively. The length of a nanostructure is approximately 10 μm, which is very beneficial in fabricating the photodetector by convenient photolithography processing. The as-fabricated Sb_2_Te_3_/Te photodetector device shows a superior photovoltaic effect because of the superior built-in electric field within the hetero-interface. The responsivity and photoconductivity are estimated as 9.5 × 10^11^ μA W^−1^ and 1.22 × 10^11^ Jones at 50 K, respectively, which is more prior than those previously reported. This study proposes that the T-shaped epitaxial Sb_2_Te_3_/Te heterojunctions show great promise for future optoelectronic device applications.

## Experimental section

### Material synthesis

An eco-friendly hydrothermal method was used to synthesize Sb_2_Te_3_/Te heterostructure nanostrings. In a typical synthesis, 2 mmol L-antimony potassium tartrate (C_8_H_4_K_2_O_12_Sb_2_, AR, 99%), 3 mmol sodium selenite (AR, 99%), and 0.3 g polyvinyl pyrrolidone (PVP, 130000, AR, 99%) were dissolved in 40 ml of ethylene glycol (AR, 99%). After vigorous stirring for 10 min, the mixture was put into a 100 ml Teflon-lined stainless-steel autoclave. The autoclave was treated at 180 °C and maintained for 48 h before being cooled in air. The precipitates were isolated by centrifugation, washed with distilled absolute ethanol and water several times to remove possible residues, and dried in a vacuum. Lastly, to improve the degree of crystallinity, the Sb_2_Te_3_/Te composite was placed in a tube and annealed to 300 °C for 2 h in argon.

### Material characterization

Morphological characterizations of the Sb_2_Te_3_/Te nanostrings were performed using scanning electron microscopy (SEM, NOVA 450, FEI) and transmission electron microscopy (TEM, G2 FEI). The crystalline structures of the as-prepared nanofibers were characterized by X-ray diffraction (XRD, Shimadzu XRD-6000). The valence state analysis of the Sb_2_Te_3_/Te nanostrings was performed with an X-ray electron spectrometer (XPS, AXIS-ULTRA DLD-600W).

### Device construction and analysis

The topological insulator Sb_2_Te_3_/Te heteronanostructures are sensitive to acetone, which is usually used to remove the photoresistor during the photolithography process. A focused ion beam (FIB) was employed to define the metal electrode during the fabrication of the NIR photodetector. Briefly, a micro-electrode on SiO_2_ (300 nm)/Si substrate was fabricated using conventional photolithography, followed by the deposition of 25 nm titanium and 35 nm gold films by high vacuum electron beam evaporation. Then, the dispersed Sb_2_Te_3_/Te nanostrings were dropped on the micro-electrode and deposited as 50 nm platinum films by FIB. The device characteristics of the topological insulator Sb_2_Te_3_/Te heterostructure are measured using a semiconductor characterization system (Keithley 4200-SCS). The test system was equipped with an automatic cooling system named CCS-350, which was a slow-temperature cycle refrigeration system. For the optoelectronic study, the 980 nm laser (CEL-PF300-T9) is employed as the monochromatic light, which is equipped with an attenuator guided to the NIR device.

## Results and discussion

**FIGURE 1:**
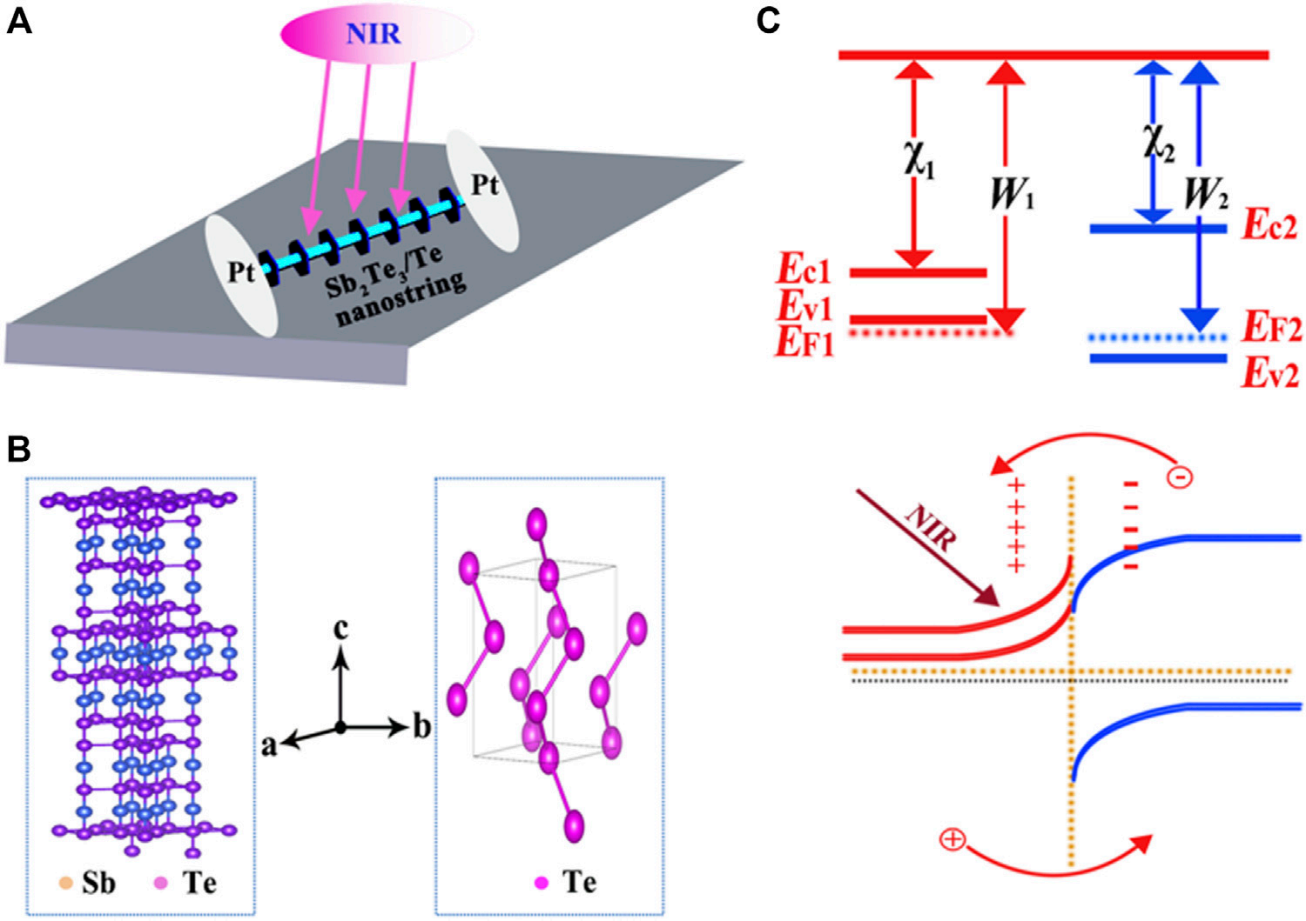
**(A)** Schematic illustration of the Sb_2_Te_3_/Te nanostring NIR photodetector. **(B)** Schematic illustration of the crystal structure of the Sb_2_Te_3_ nanosheet and Te nanowire. **(C)** Energy band diagram of the Sb_2_Te_3_/Te nanostring photodetector without and with NIR light illumination.

The proof-of-concept photodetection device (Figure 1A) based on Sb_2_Te_3_/Te nanostrings was fabricated on an SiO_2_ substrate and the trench width was approximately 5 μm. The Te nanowire is separated by a periodically arranged Sb_2_Te_3_ nanoplate, which formed distinctive p-p heterojunctions. The unique nanostring structure leads to a higher photoelectric conversion efficiency. Figure 1B shows the crystal structure models of Sb_2_Te_3_ and Te. The Sb_2_Te_3_ crystal structure consists of approximately five-atom layers along the *c*-direction, which are known as quintuple layers. Each layer consists of five atoms in order as follows: Te1–Sb–Te2–Sb0–Te10. Furthermore, Te is a hexagonal crystal formed by the accumulation of helical chains through van der Waals interactions. The band structures of Sb_2_Te_3_ and Te are depicted in Figure 1C, respectively. Sb_2_Te_3_ is a p-type topological insulator with a Femi level located in the valance band. The Te is also a p-type semiconductor whose band gap is larger than Sb_2_Te_3_. After combination, a heterojunction can be formed at the interface between Sb_2_Te_3_ and Te. As the work function of Sb_2_Te_3_ is smaller than Te, its electrons will flow into Te and this charge transfer process will form a built-in potential field at the interface. Under infrared light illumination, electron-hole pairs can be generated in Sb_2_Te_3_ and holes will be transferred to Te by built-in potential. These carrier generations and transfers will lead to a detectable photocurrent.

**FIGURE 2:**
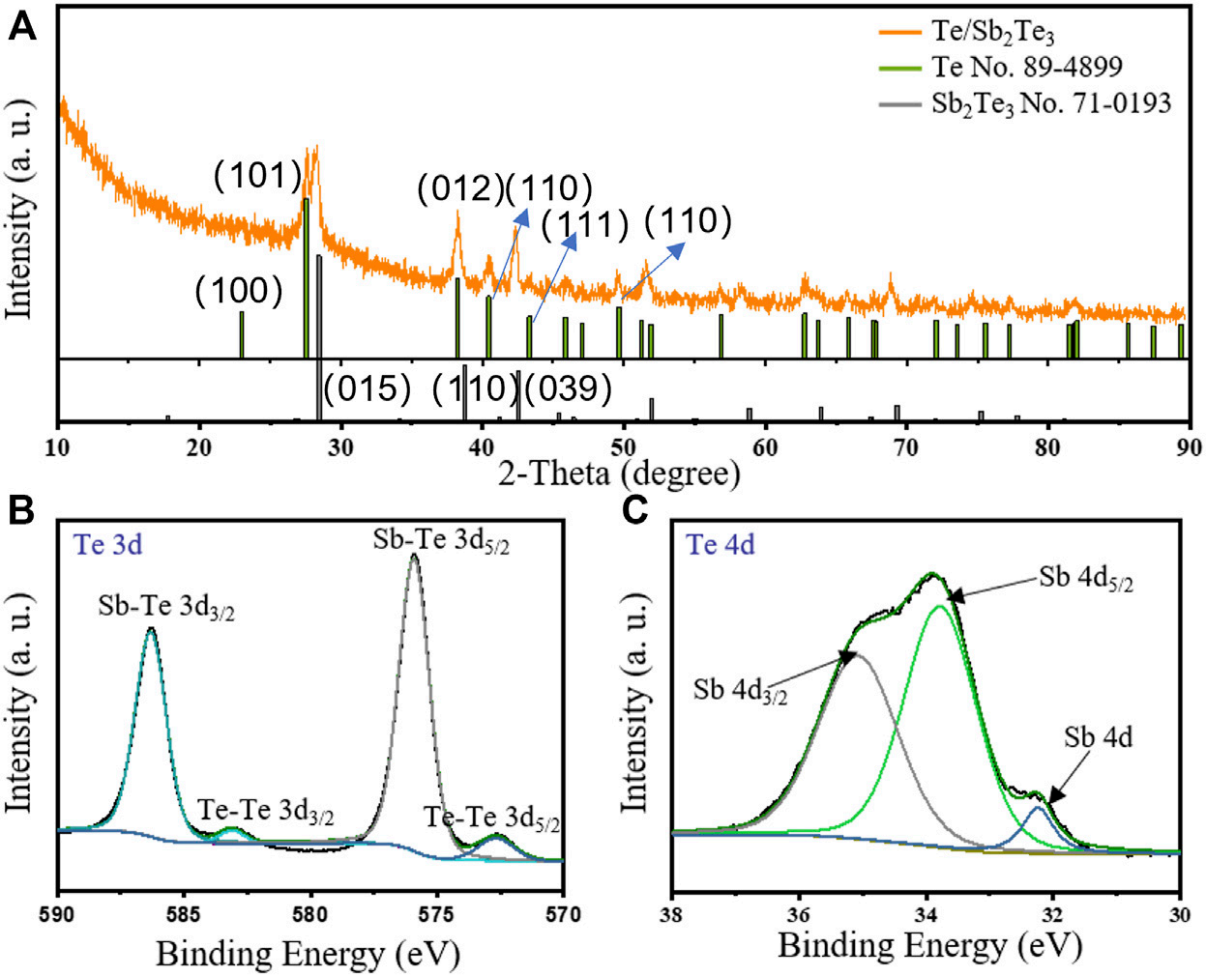
**(A)** X-ray diffraction (XRD) pattern of the Sb_2_Te_3_/Te nanostrings. **(B,C)** XPS spectra of Te (3d) and Sb (4d).

The Sb_2_Te_3_/Te nanostrings are characterized by XRD. Figure 2A (turquoise line) shows the XRD pattern of the Sb_2_Te_3_/Te nanostrings. All peak positions in Figure 2A are indexed to the rhombohedral Sb_2_Te_3_ (JCPDS No. 71-0393) and the hexagonal Te (JCPDS No. 89-4899). The characteristic peaks of Sb_2_Te_3_ and Te are exhibited in Figure 2A such as (015), (110), (205), (101), (012), and (110) planes, which suggest that the as-prepared product is composed of rhombohedral Sb_2_Te_3_ and hexagonal Te. In addition, the diffraction intensities of Sb_2_Te_3_ (006) and (1010) planes are extremely sharp; however, those of the (101) and (107) planes are distinctly weak, showing that the (hk0) planes in the Sb_2_Te_3_ nanostructure grew faster than the (*hkl*, *l* ≠ 0) planes. Therefore, the Sb_2_Te_3_ crystallization is preferentially grown along with the *a* or *b* axle instead of the *c* axle. As a result, ultrathin Sb_2_Te_3_ nanosheets can be generated in the final products. It is remarkable that the crystallinity is scored as 91.67% after refining (Supplementary Figure S1).

The X-ray photoelectron spectroscopy (XPS) spectra further clarify the structure of Sb_2_Te_3_/Te nanostrings. The sharp peaks of Te (3d) and broad peaks of Sb (3d, 4d) can be clearly separated (Supplementary Figure S2). Figures 2B and C reveal that the electron-binding energies of Te 3d_3/2_ and Te 3d_5/2_ located at 586.3 eV and at 575.9 eV, respectively, which corresponds to the valence of *Sb-Te*. The binding energies located at 583.0 and 572.6 eV can be ascribed to the 3d_3/2_ and 3d_5/2_ of *Te-Te* valence. The Raman spectra can also confirm the existence of Te and Sb_2_Te_3_. Two typical characteristic peaks are located at 179 and 235 cm^−1^, which can actually be assigned to Te (Yin et al., 2018) (Supplementary Figure S3). The representative signals of Sb_2_Te_3_ are located at 309 and 343 cm^−1^. All facts confirm that the as-synthesis product exhibits a fine crystallinity with definite constitution and structure.

**FIGURE 3:**
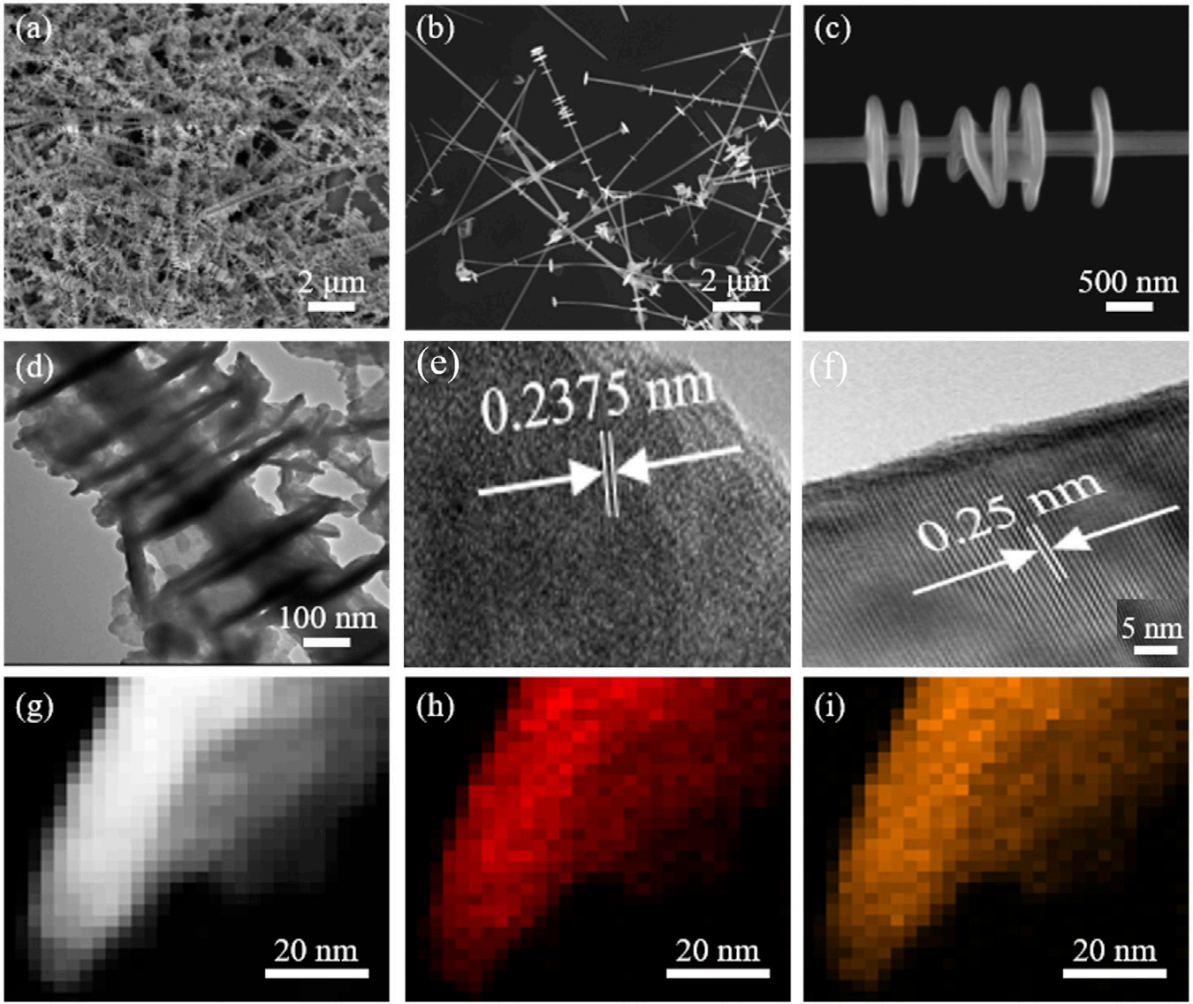
**(A–C)** SEM images of the Sb_2_Te_3_/Te nanostrings at different magnifications. **(C)** TEM image of the Sb_2_Te_3_/Te nanostrings. **(D)** TEM image of the Sb_2_Te_3_/Te nanostrings. **(E,F)** HRTEM images of the Sb_2_Te_3_/Te nanostrings **(G–I)** and elemental mapping images of Sb_2_Te_3_/Te nanostrings.

Figures 3A–C reveal the morphologies of the nanostrings at different magnifications by field-emission scanning electron microscopy (FESEM). The nanostrings are composed of multiple nanosheets that are strung together through the center by Te nanowires. The length of the Te nanostructure is approximately 10 μm (Supplementary Figure S4), which is beneficial for the photodetection device fabrication by conventional photolithography. Figure 3D demonstrates that the Sb_2_Te_3_ nanosheets are embedded in the Te nanowire and the diameter is approximately 300 nm. HRTEM pattern analysis effectively indicates the monocrystalline texture of Sb_2_Te_3_/Te nanostrings (Figures 3E and F). The lattice fringes are noticeable and the d-spacings are 0.2375 and 0.25 nm, which correspond well to the Tellurium (012) and Sb_2_Te_3_ (110) lattice planes, respectively. The selected area electron diffraction (SAED) pattern (Supplementary Figure S5) is well indexed to the hexagonal phase of Tellurium, and corresponding to the diffraction peaks of (012), (101), and (110) planes shown in the XRD pattern. Energy dispersive spectrometer (EDS) spectra confirmed that the nanostrings are composed of Sb and Te elements (Figures 3G–I). Figures 3G–I exhibit the TEM elemental mapping images of the Sb_2_Te_3_/Te nanostring, which further confirms that the as-prepared product is only composed of Te and Sb elements.

**FIGURE 4:**
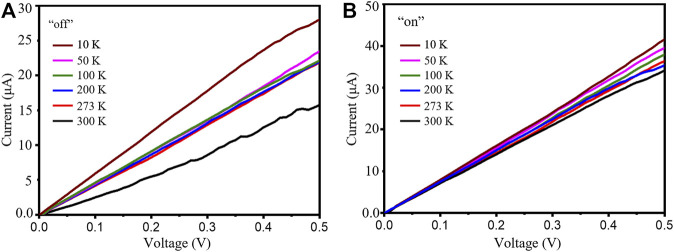
I-V curves of the Sb_2_Te_3_/Te nanostrings in the dark **(A)** and under illumination **(B)** at various temperatures from 10 to 300 K.

The temperature-dependent current-voltage (I-V) curves of the as-fabricated device are studied to disclose its electrical transportation characteristics. Figures 4A and B demonstrate the I-V characteristics from 10 to 300 K in the dark and under 980 nm light illumination (0.5 mW cm^−2^). The dark current increases with falling temperature. This can be attributed to the unique electronic construction, particularly when the Fermi level is close to the Dirac point. The I-V curves are virtually linear when illuminated by 980 nm light and show that the Te nanowire and Pt-Ti/Au electrode can form a contact form with Ohmic contact.

**FIGURE 5:**
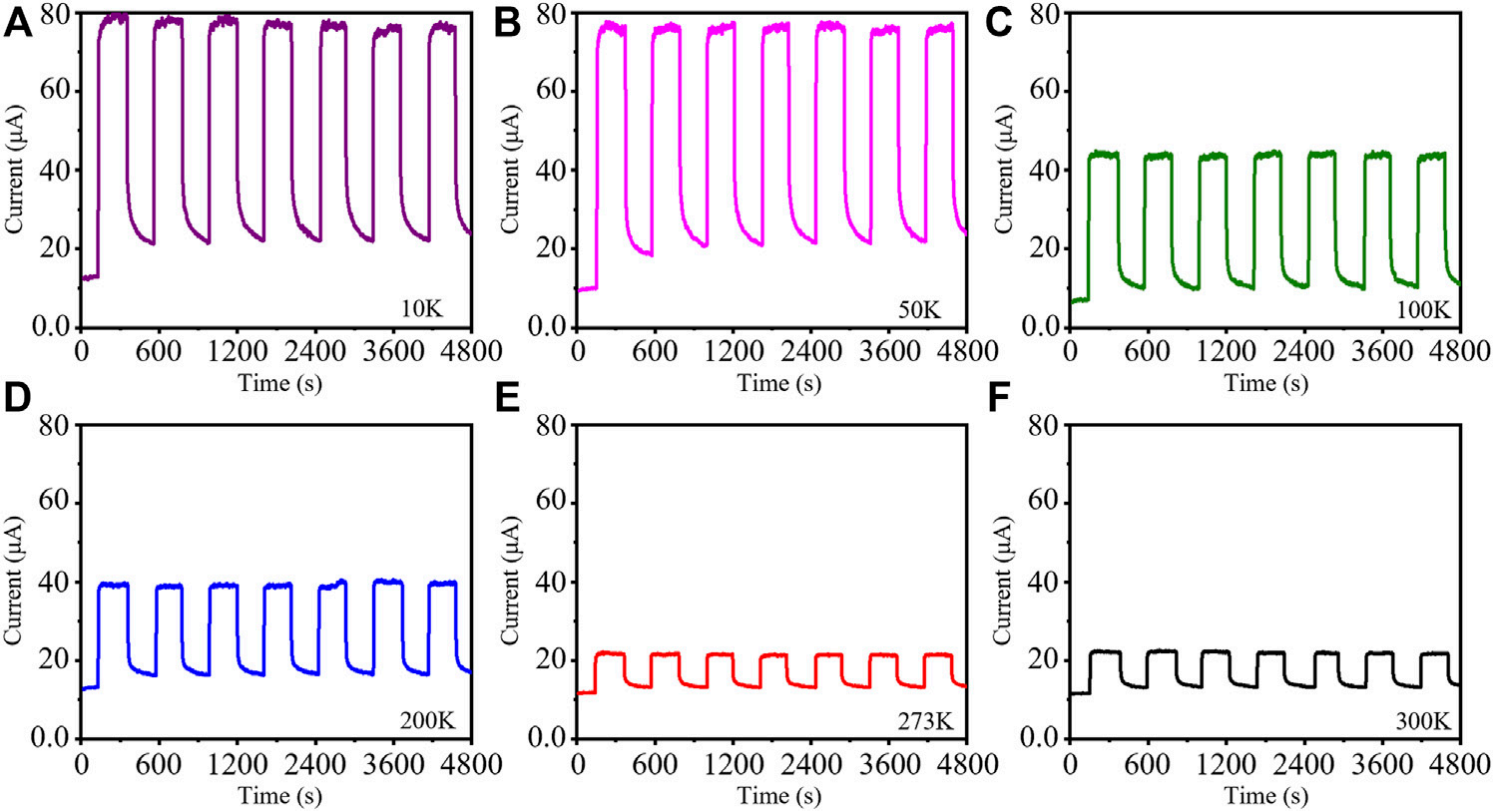
Time-resolved photoresponse of the Sb_2_Te_3_/Te nanostring photodetector under different temperatures at 1 V bias voltage: **(A)** 10 K, **(B)** 50 K, **(C)** 100 K, **(D)** 200 K, **(E)** 273 K, and **(F)** 300 K.

Considering the temperature-dependent electrical properties of the Sb_2_Te_3_/Te nanostrings, the photoresponse characteristics at different temperatures were studied. Figure 5 shows the temporal photoresponse properties of the device at 10K, 50K, 100K, 200K, 273K, and 300K with periodic irradiation of 980 nm at a bias voltage of 1 V. The figure shows that when the bias voltage is kept unchanged, the change of the current is consistent with the changes of the temperatures. The dark current can decrease to the lowest value of 8.5 μA at 100K and the photocurrent can reach its highest value of 79.5 μA at 10K under the 1 V bias voltage and on/off illumination of 980 nm light. Additionally, the photoresponse characteristics of the as-constructed photodevice are homologous at temperatures of 273 and 300 K due to the temperature effect (Qi et al., 2018). The optical switching behavior of this photodetector is highly reversible with good stability and reproducibility. There is no significant degradation in its switching behavior even after multiple periodic optical switching changes. It is worth noting that different from the conventional photodetectors with a very fast response speed based on semiconductor nanostructures, the as-fabricated device has a moderately (several hundred seconds) slow rising and falling time (Cai et al., 2022). The difference in the response speed is possible due to their distinction of the band structures.

**FIGURE 6:**
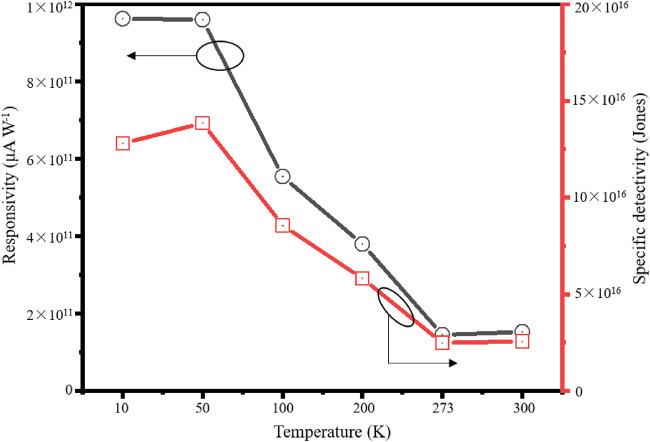
Responsivity (*R*) and detectivity (D*) of a selenium self-supporting film photodetector under different light intensities.

The responsivity ® and detectivity (D*) are also two key index factors of photodetectors. “R” is the photocurrent per unit incident light power on the calculating device, which can reflect the sensitivity of the device to the intensity of incident light. “D*” shows the performance of detecting weak light (Yu et al., 2018). These two key parameters can be derived according to the following equations:

$$R=\frac{I_{ph}-I_{d}}{PS}$$ (1)

$$D^{*}=\frac{RS^{1/2}}{{\left(2eI_{d}\right)}^{1/2}}$$ (2)

where *I*_*ph*_ is the photocurrent, *I*_*d*_ is the dark current, *P* is the incident light power density (0.5 mW cm^2^), *S* is the effective area of the device receiving light (∼1.5 × 10^−8^ cm^2^), and *e* is the fundamental charge (1.6 × 10^−19^ C) (Xu et al., 2022; Zhang et al., 2022). According to Eqs 1, 2, the “R” and “D*” of the detector under 3 V bias voltage and different illumination intensities are shown in Figure 6. The results show that the detector has a photoresponsive®(R) of approximately 9.5 × 10^11^ μA W^−1^ and a detectable rate (D*) of approximately 1.3×10^17^ Jones under 1 V bias voltage and 50 K with the light irradiation of 980 nm.

## Conclusion

We report a near NIR photodetector based on a topological insulator antimony telluride (Sb_2_Te_3_) and tellurium (Te) heterostructure, which are prepared by controllable hydrothermal and photolithography methods. The elaborately constructed device exhibits topological insulator properties, and the resistance especially decreases with increasing temperature in the range of 10–300 K. Further optoelectronic characterization shows that the as-fabricated photodetector delivers obvious sensitivity to 980 nm light illumination. The performance of responsivity and detectivity are remarkable and are much better than those of other Te-based topological insulator photodetector devices. The research suggests that the as-constructed NIR photodetector may have great potential in low-temperature optoelectronic devices.

## Data Availability

The datasets presented in this study can be found in online repositories. The names of the repository/repositories and accession number(s) can be found at: https://www.ccdc.cam.ac.uk/structures/- 2211392 and 2211393.
