# Supplementary material for: Design of Sb2Te3 nanoblades serialized by Te nanowires for a low-temperature near-infrared photodetector
Source: Front Chem. 2022 Nov 18;10:1060523. doi: 10.3389/fchem.2022.1060523 (PMC9716093; doi:10.3389/fchem.2022.1060523)
Supplement: Supplementary file 1 [file DataSheet1.doc]

**Design of** **Sb_2_Te_3_** **nanoblades serialized by Te nanowires for low-temperature near infrared photodetector**

Hong Yin^a, b, *^, Huaiyu Li^a^, Xiang-xiang Yu^d^, Minglei Cao^c,*^

^a^School of Chemistry and Chemical Engineering, Hunan Institute of Science and Technology, Yueyang, 414006, China

^b^International Iberian Nanotechnology Laboratory (INL), Av. Mestre Jose Veiga, 4715-330 Braga, Portugal

^d^School of Physic and Optoelectronic Engineering, Yangtze University, Jingzhou 434023, China

*Corresponding Author.

E-mail: 2017507027@hust.edu.cn (H. Yin).

cml07114052@163.com ( ML. Cao)


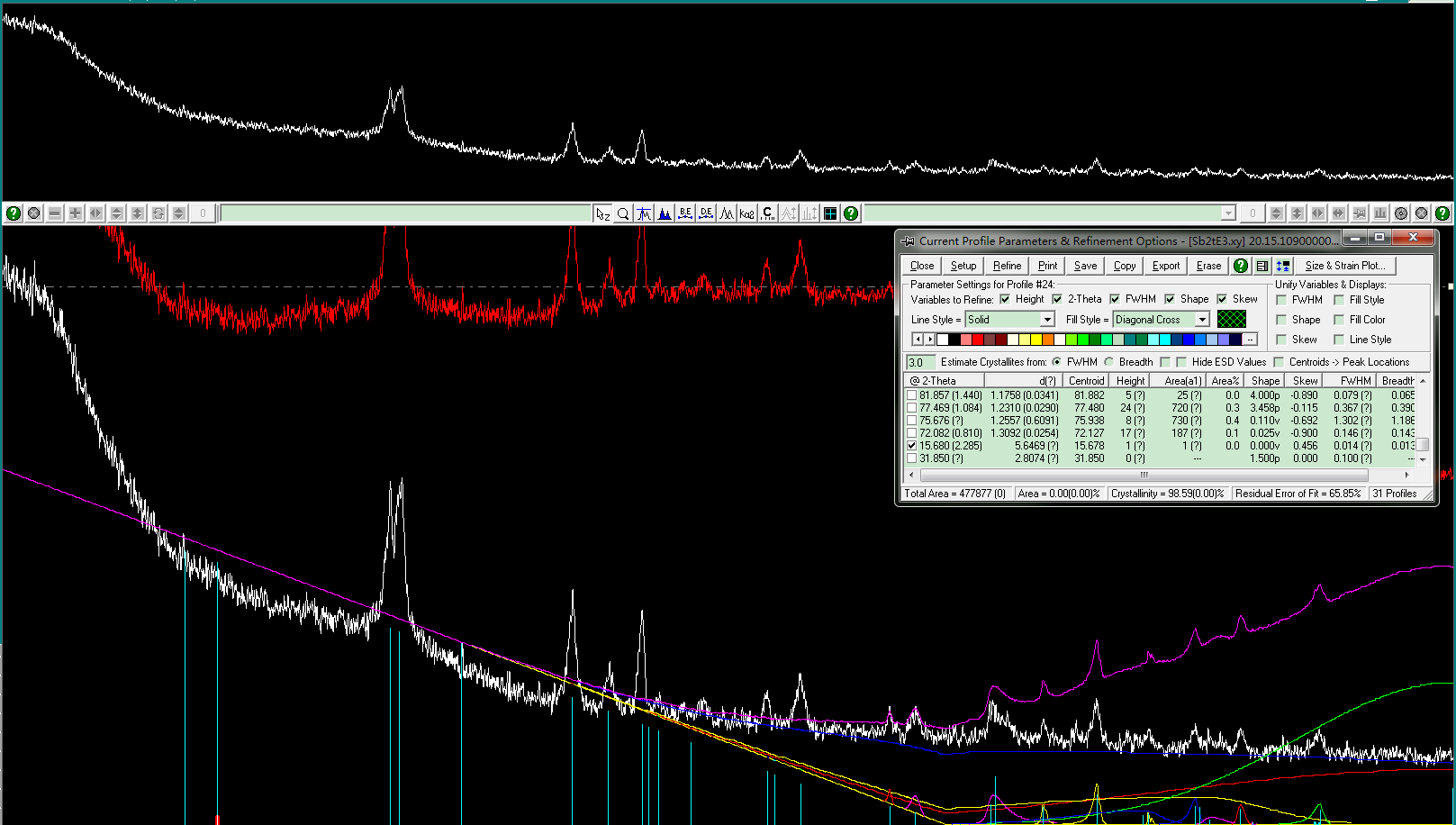


**Figure S1** The refined XRD patterns of STNH material.


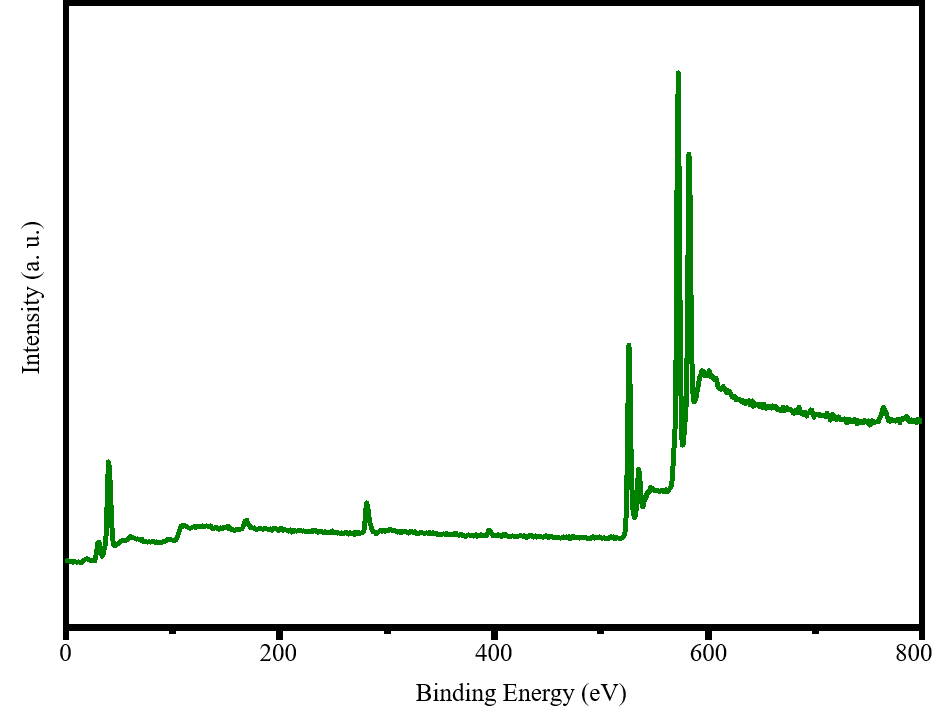


**Figure S2** The XPS spectrum of the Sb_2_Te_3_/Te nanostrings.


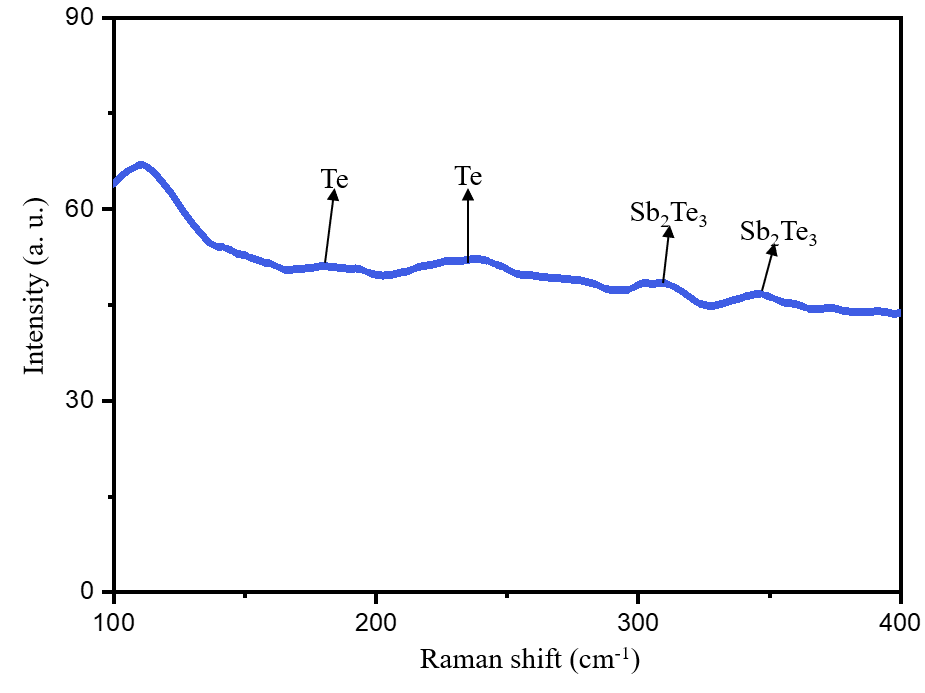


**Figure S3** Raman spectra of the as-prepared Sb_2_Te_3_/Te nanostrings.

**
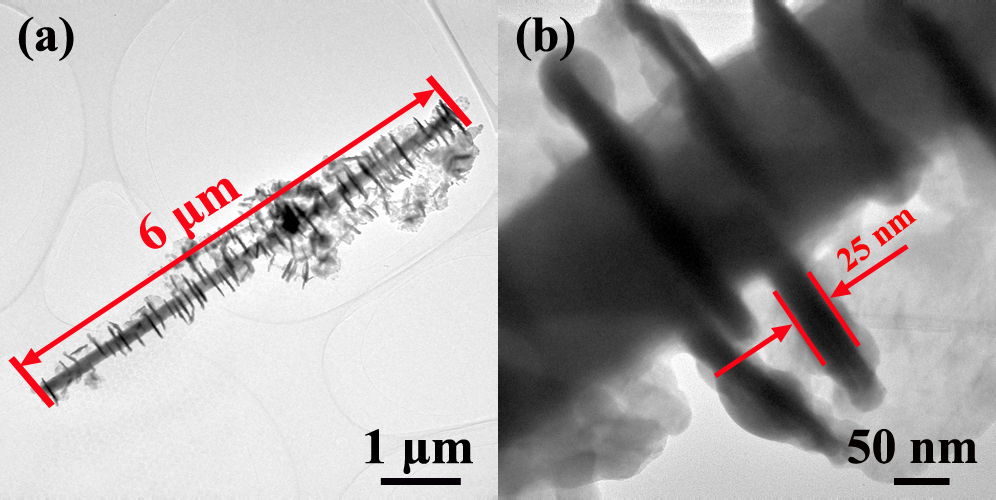
**

**Figure S4** (a) The length of as-prepare STNH material. (b) The thickness of as-prepared Sb_2_Te_3_/Te nanostrings.


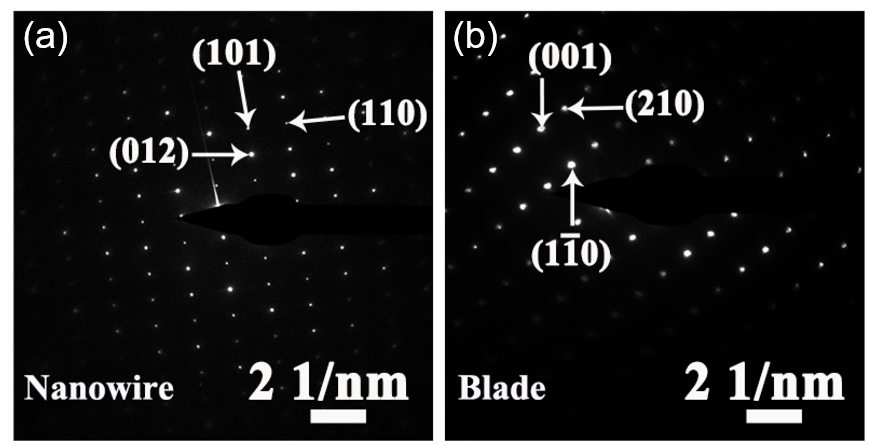


**Figure S5** (a) and (b) SAED patterns of Te nanowire and Sb_2_Te_3_ nanoblade, respectively.

Where *I*_p_, *I*_d_, *P*_opt_, *η*, *q*, *λ*, *h*, *c, P* and *S* are the photocurrent, the current without light illuminated on, the power of the light, the quantum efficiency (for convenience, assuming *η* = 1), the absolute value of electron charge (1.6 × 10^-19^ Coulombs), the wavelength of illuminated light (980 nm), the Planck’s constant (6.626 × 10^-34^ J·s), the velocity of light (3 × 10^8^ ms^-1^), the optical power density and radiating surface respectively.
